# Considering User Experience and Behavioral Approaches in the Design of mHealth Interventions for Atrial Fibrillation: Systematic Review

**DOI:** 10.2196/54405

**Published:** 2024-10-04

**Authors:** Sagar Suresh Kumar, Patricia Connolly, Anja Maier

**Affiliations:** 1 Department of Design, Manufacturing and Engineering Management (DMEM) University of Strathclyde Glasgow United Kingdom; 2 Department of Biomedical Engineering University of Strathclyde Glasgow United Kingdom

**Keywords:** atrial fibrillation, wearable devices, lifestyle modification, user experience, design for behavior change, systems thinking, cardiac disease, stroke, heart disease, complication, mobile health, systematic review, usability, mHealth, intervention

## Abstract

**Background:**

Atrial fibrillation (AF) is a leading chronic cardiac disease associated with an increased risk of stroke, cardiac complications, and general mortality. Mobile health (mHealth) interventions, including wearable devices and apps, can aid in the detection, screening, and management of AF to improve patient outcomes. The inclusion of approaches that consider user experiences and behavior in the design of health care interventions can increase the usability of mHealth interventions, and hence, hopefully, yield an increase in positive outcomes in the lives of users.

**Objective:**

This study aims to show how research has considered user experiences and behavioral approaches in designing mHealth interventions for AF detection, screening, and management; the phases of designing complex interventions from the UK Medical Research Council (MRC) were referenced: namely, identification, development, feasibility, evaluation, and implementation.

**Methods:**

Studies published until September 7, 2022, that examined user experiences and behavioral approaches associated with mHealth interventions in the context of AF were extracted from multiple databases. The PRISMA (Preferred Reporting Items for Systematic reviews and Meta-Analyses) guidelines were used.

**Results:**

A total of 2219 records were extracted, with only 55 records reporting on usability, user experiences, or behavioral approaches more widely for designing mHealth interventions in the context of AF. When mapping the studies onto the phases of the UK MRC’s guidance for developing and evaluating complex interventions, the following was found: in the identification phase, there were significant differences between the needs of patients and health care workers. In the development phase, user perspectives guided the iterative development of apps, interfaces, and intervention protocols in 4 studies. Most studies (43/55, 78%) assessed the usability of interventions in the feasibility phase as an outcome, although the data collection tools were not designed together with users and stakeholders. Studies that examined the evaluation and implementation phase entailed reporting on challenges in user participation, acceptance, and workflows that could not be captured by studies in the previous phases. To realize the envisaged human behavior intended through treatment, review results highlight the scant inclusion of behavior change approaches for mHealth interventions across multiple levels of sociotechnical health care systems. While interventions at the level of the individual (micro) and the level of communities (meso) were found in the studies reviewed, no studies were found intervening at societal levels (macro). Studies also failed to consider the temporal variation of user goals and feedback in the design of long-term behavioral interventions.

**Conclusions:**

In this systematic review, we proposed 2 contributions: first, mapping studies to different phases of the MRC framework for developing and evaluating complex interventions, and second, mapping behavioral approaches to different levels of health care systems. Finally, we discuss the wider implications of our results in guiding future mHealth research.

## Introduction

### The Chronic Condition of Atrial Fibrillation and the Goal of Clinical Interventions

Atrial fibrillation (AF) is one of the leading heart diseases in today’s world and affects more than 0.5% of the global population. It is caused by irregular pulses in the heart atria, which leads to an increased risk of stroke, heart failure, mortality, and other poor outcomes [1]. The integrated model of multimorbidity and symptom science attributes the progression of chronic conditions, such as AF, to the interplay of various factors across the following 3 domains: risk factors, interventions or conditions or symptoms, and outcomes [2]. AF has both nonmodifiable risk factors, such as age and genetics, as well as several modifiable and lifestyle-based risk factors, such as hypertension, alcohol, and obesity, which account for 30% to 40% of the risk of developing AF [3]. These risk factors can also lead to the co-occurrence of other diseases, such as myocardial infarction, thereby worsening health outcomes [4]. The success of chronic disease interventions lies in their ability to impede or reverse the transition of risk factors to poor patient outcomes through different means [2].

### Challenges Faced by Existing AF Clinical Interventions

In the case of AF, early detection and subsequent management through catheter ablation and other interventions can prevent up to 97% of cases from progressing to persistent and permanent forms [5], which are associated with poorer health outcomes, as well as increased long-term costs [6]. However, up to 40% of early cases may be asymptomatic and episodic or paroxysmal, that is, they may not be able to be detected through traditional diagnostic interventions such as routine checkups. Instead, they require continuous monitoring outside of hospitals and other traditional care environments [7]. Moreover, traditional clinical guidelines and their concomitant interventions mostly do not consider the different challenges faced by patients in their respective journeys and daily lives in their target outcomes [8]. The burden of AF goes beyond physical outcomes and also takes a huge toll on the social well-being of patients, by impeding their ability to perform day-to-day activities, affecting their fulfillment of social roles, and burdening family members and caregivers [9]. AF prevalence is expected to increase by more than 60% in the next 30 years. As the condition affects up to 17% of people older than 80 years, the increase in life expectancy, as well as the global population, is expected to pose significant social and economic challenges to the world, especially in middle- and high-income countries [1]. In the United Kingdom, AF care currently accounts for 0.9% to 1.6% of the annual expenditure of the National Health Service and is expected to increase to up to 4.27% over the next 2 decades [10]. The National Health Service has been overwhelmed due to the growing demand for cardiovascular care, with the number of people at the end of 2022 waiting for a heart-related treatment increasing by 280 times compared to before the pandemic [11].

### The Potential for Mobile Health in Alleviating Said Challenges

In this regard, mobile health (mHealth) devices consisting of wearable devices and apps can alleviate this inaccessibility of care and workforce constraints by offering alternative and personalized interventions to support care services in the detection and management of chronic conditions [12]. In the detection of AF, different sensing modalities, such as electrocardiography (ECG) [13] and photoplethysmography (PPG) [14], have been integrated within wearable devices, such as smartwatches and smartphones, to facilitate continuous monitoring, which is essential in detecting paroxysmal and asymptomatic cases. Mobile apps can aid patients in improving their lifestyles and reducing risk factors, which in conjunction with regular care can improve recovery, as well as reduce symptomatic burden [15]. For example, a 10% weight reduction in individuals with obesity can result in a 27% reduction in the risk of AF recurrence following catheter ablation [16]. Apps can also assist patients in managing AF by improving medication adherence, increasing their knowledge, improving patient-physician interactions, and so on [17-19].

### The Need to Consider User Experiences and Usability of mHealth Interventions

While mHealth technology may have promising potential to improve AF care [20], we need to go beyond its potential clinical benefits and also consider the experiences and perspectives of patients, as well as the implications of introducing such interventions in people’s lives. Emotions and feelings experienced by patients as a result of their diagnosed conditions can greatly impact the usability of interventions [21]. Additionally, environmental and individual risk factors can impact an individual’s emotional comfort, which is defined as a state of well-being where patients are satisfied with the role of clinical intervention in improving their respective journeys in life [22]. In other chronic disease contexts, patients’ emotional comfort with regard to mobile and digital interventions has included various aspects of their daily lives such as diet, physical activity, work, and social occasions [21]. Emotionally comfortable patients are more likely to cooperate with care, thereby increasing the usability of services and interventions [22]. As per International Organization for Standardization (ISO) standard ISO 9241-11:2018—ergonomics and human-system interaction, usability is defined as: “the extent to which a system, product, or service can be used by specified users to achieve specified goals with effectiveness, efficiency, and satisfaction in a specified context of use.” A user is defined as an individual who interacts with the device of interest [23].

### The Lack of Review Papers Considering User Experiences for mHealth Interventions

Currently, there is a lack of systematic reviews that analyze the extent to which patient experiences have been considered in the design of “usable” mHealth interventions for the detection and management of AF. Reviews to date have mostly focused on technical aspects of devices and apps such as accuracy, diagnostic rate, readability, and quality [24-27]. In other contexts, user-centered approaches entailing different co-design methods to examine the needs, perspectives, and overall experiences of patients have been used for the design of more usable health care interventions [28]. For example, in the case of wearable technology to monitor the activity and mobility of patients with dementia, direct observation, questionnaires, and interviews based on a technology adoption model, that is, the Unified Theory of Acceptance and Use of Technology, have yielded positive outcomes. Patients found the ability of such interventions to provide navigation support useful for going out of their homes without the fear of getting lost. They also felt that its benefits had a carryover effect on other aspects of their life including personal autonomy and relationships [29,30]. The discourse in the *JMIR Human Factors* has also focused on reviewing and consolidating the evidence on the usability of mHealth interventions for different chronic conditions [31-33]. Hence, the critical need for a systematic review to ascertain the extent to which user-centered approaches have been embedded in the design of mHealth interventions for AF.

### Contextual Interactions Between AF mHealth Interventions and User Experiences of the Wider Health System

When designing mHealth interventions for AF, contextual interactions with the wider health care system [34] need also to be considered as they can further shape experiences and perspectives. This may include the expertise required by those receiving and delivering the intervention [34]; the people involved, for example, patients, carers, and health care professionals; as well as influences through technology, facilities, or regulations [35]. Health care professionals would need to work closely with mHealth devices and apps, and the introduction of new technology impacts clinical workflows, for example, by easing or increasing daily workload [36] and as such impacting human behavior.

Behavior is closely related to user experiences; it can drive as well as be the target for mHealth interventions. Two examples from the studies reviewed are highlighted: in the first case, a user’s experience can influence their behavior with regard to technology acceptance and the uptake of interventions. For example, a user’s concern about a device and its data causing them unnecessary worry and anxiousness can lead to its rejection [37], and varying levels of adherence to self-monitoring interventions have been reported in the past [38,39]. Preferences regarding the use of mHealth can also vary among different user groups [40]. In the second case, interventions aim for a behavioral or lifestyle modification, where healthier habits are sought to minimize risk factors associated with the condition, and thus, enhance users’ experiences in life [41,42]. While studies to date have explored the incorporation of behavior change techniques [43] in wearable devices and mHealth more widely, the focus has not been on AF in particular [44-46]. Furthermore, behavioral interventions can be classified based on the level of the sociotechnical health system they operate within [47]. At the micro level, interventions focus on the individual and their characteristics including knowledge, attitudes, beliefs, or personality traits. Meso- and macro-level approaches are at the level of groups and whole societies, respectively [46]. Long-term behavioral change approaches, which would be necessary given the chronic nature of AF, also need to account for the following: first, the practical limitations of conducting long-term validation studies, and second, as discussed above, the need to address the wider context and complexity of interactions within health care systems [43]. Finally, a certain level of digital literacy and expertise would be required to use such wearable devices and apps [26]. Therefore, AF mHealth interventions can be regarded as complex.

### The New Framework for Developing and Evaluating Complex Interventions

Following the new framework commissioned by the National Institute of Health Research and the UK Medical Research Council (MRC) 2021, the design of complex interventions unfolds in the following phases: identification, development, feasibility, evaluation, and implementation [34]. In the identification and development phases, an existing intervention may be adapted for a new context, or it may be developed from scratch. The feasibility testing phase analyses whether the intervention can meet certain intended outcomes. The evaluation phase goes beyond just assessing whether the intervention works and ascertains what other impacts it has and how it interacts in the context in which it is implemented. In the implementation phase, the results of the previous phases are collated to determine steps to facilitate real-world adoption of the intervention in different health care systems. A wide range of other health care interventions ranging from new surgical procedures to redesigning entire health care programs have been successfully realized as complex interventions. For example, the Links Worker program in Glasgow, Scotland, sought to link people in primary care with community resources targeted individual (micro) and groups and communities (meso) levels and assessed multiple measures including bereavement, substance use, employment, and learning difficulties [34].

### The Need to Consider User Experiences and Behavioral Approaches in Designing Complex mHealth Interventions

Previous studies entailing mHealth technology for other apps besides AF have failed to address the potential complexity of health system interactions, and thus, have struggled to transition from pilot studies to interventions actively used in health care systems. The reasons attributed range from various issues including behavioral resistance from patients and health care professionals, poor digital literacy surrounding mHealth use, and a lack of interoperability between devices and systems [36]. On the whole, interventions need to be designed to help users reach their respective goals in their journeys, that is, especially social empowerment for patients [9] and workflow integrability for health care professionals [36]. As such, in this review study, we aimed to ascertain the extent to which studies to date have considered user experiences and related behavioral approaches in different phases of the design of usable complex AF mHealth interventions.

## Methods

The systematic review was conducted as per the PRISMA (Preferred Reporting Items for Systematic Reviews and Meta-Analyses) guidelines [48].

### Data Sources and Search Strategy

#### Overview

The following databases were searched: PubMed, Cochrane, Scopus, Web of Science, and Embase Ovid. The search was not restricted to any particular time period. Only papers in English were considered. The search string used the terms and logic shown in Textbox 1.

**Search strings.**

- For PubMed, Cochrane, and Web of Science
  (usefulness OR usability OR utility OR use OR “user experience” OR “user-experience”) AND (monitoring OR “remote care” OR detection OR management OR treat* OR screening OR assess* OR diagnosis OR “lifestyle modification” OR “lifestyle change” OR “integrated care” OR behavior* OR behaviour* OR “weight loss” OR “risk factor”) AND (wearable OR “emerging technology” OR “health technology” OR “health device” OR “mobile health” OR mHealth OR “handheld ECG” OR smartwatch OR band OR “ECG patch” OR “implantable loop recorder” OR PPG OR smartphone OR phone OR accelerometer OR gyroscope) AND (“atrial fibrillation” OR “atrial arrhythmia”).
- For Scopus
  (TITLE-ABS-KEY (usefulness OR usability OR utility OR “use” OR “user experience” OR user-experience) AND TITLE-ABS-KEY (monitoring OR “remote care” OR detection OR management OR treat* OR screening OR assess* OR diagnosis OR “lifestyle modification” OR “lifestyle change” OR “integrated care” OR behavior* OR behaviour* OR “weight loss” OR “risk factor”) AND TITLE-ABS-KEY (wearable OR “health technology” OR “emerging technology” OR “health device” OR “mobile health” OR mHealth OR “handheld ECG” OR smartwatch OR band OR “ECG patch” OR “implantable loop recorder” OR ppg OR smartphone OR phone OR accelerometer OR gyroscope) AND TITLE-ABS-KEY (“atrial fibrillation” OR “atrial arrhythmia”)).
- For Embase Ovid
  usefulness OR usability OR utility OR (user experience) OR user-experience AND (remote care) OR detection OR management OR treat* OR screening OR assess* OR monitoring OR diagnosis OR (lifestyle modification) OR (lifestyle change) OR (integrated care) OR behavior* OR behaviour* OR (weight loss) OR (risk factor) AND (health technology) OR (emerging technology) OR (health device) OR (mobile health) OR mHealth OR (handheld ECG) OR (ECG patch) OR (implantable loop recorder) OR smartwatch OR band OR PPG OR wearable OR smartphone OR phone OR accelerometer OR gyroscope AND (atrial fibrillation) OR (atrial arrhythmia).

### Study Selection and Extraction

Studies that were published before September 7, 2022, and met the criteria described in Textbox 2 were selected. The abstracts of the records were reviewed in the first round of literature screening. The aims, methodology, and results of selected papers were assessed after extracting the full-text records.

**Study inclusion and exclusion criteria.**

**Inclusion criteria**

- At least 1 group of patients with atrial fibrillation (AF) received a device, app, or other mobile health (mHealth) intervention
- Studies must consider the usability of the mobile intervention through user experiences, behavior, or some other criteria in their study outcomes in at least 1 phase of the complex intervention design process
- The abstracts of the records were reviewed in the first round of literature screening. The aims, methodology, and results of selected papers were assessed after extracting the full-text records
- Papers published in English

**Exclusion criteria**

- Did not entail AF or an mHealth intervention
- Did not consider the usability of the intervention in any of the design stages
- Could not access full-text records
- Papers in other languages

## Results

### Overview

**Figure 1:**
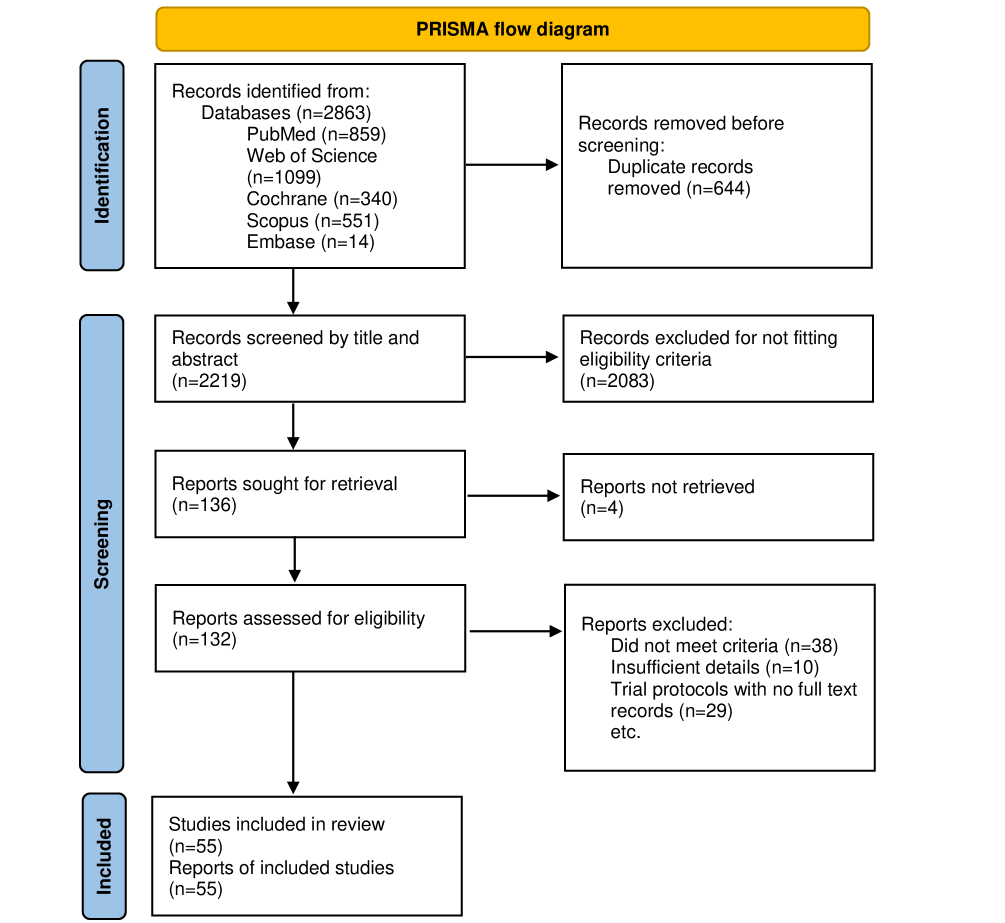
PRISMA (Preferred Reporting Items for Systematic Reviews and Meta-Analyses) flow diagram: systematic review procedure and the resulting number of relevant papers.

Among the 2219 records identified, with only 55 records met the full inclusion criteria. The results of the systematic literature review are shown in Figure 1.

The interventions in the studies selected based on their application can be classified into 3 types of interventions: detection, screening, and management. The studies focused on the experiences and behavior of users with respect to the focus areas described in Table 1. Most of the studies that focused on the detection of AF using mHealth devices did not specify the type of AF. Biersteker et al [39] proposed an intervention to detect AF postcardiac surgery while Nguyen et al [49] proposed a smartphone-based device to detect pediatric AF and other arrhythmias. A variety of devices, including smartwatches and smartphones, that incorporated ECG, PPG, and other sensors were used to continuously support patients in detecting AF. Some studies aimed at screening, that is, the use of mHealth for the mass diagnosis of AF among large groups of people for different environments, age groups, and risk factors. Orchard et al [50] studied screening in a general practice setting [51-53] and a routine vaccination setting. Macniven et al [54] assessed screening among Aboriginal communities in Australia. Apps for managing AF focused on improving the knowledge of patients, medication adherence, clinical interactions, clinical decision support, and other applications are listed in Table 1. As shown in Table 2, most of the studies (43/55, 78%) considered the user in the feasibility phase through customized or validated usability questionnaires. Some studies have considered multiple phases and types of interventions [34].

**Table 1:** The different types of interventions in the selected studies and their focus areas for assessing user experiences and behavioral approaches.

Types of interventions and focus areas			Specific focus areas and examples
**Detection**			
	Device type	Smartphones [14,38,49,55-59] Smartwatches and Bands [56,60-63] Handheld recorders [39,63-67] Implantable monitors [68,69]	
	AF^a^ type	Postoperative [58] Pediatric [49]	
**Screening**			
	Environments	Pharmacy [70] Aboriginal communities [54,71] Metropolitan care [52,53] Rural care [51] Influenza vaccinations [50]	
	Patient characteristics	Age [72-74] Other risk factors [72]	
**Management**			
	Application	Patient education [13,14,17-19,57,63,64,66,75-81] Health worker education [82,83] Clinical decision support [84] Medication adherence [17,81,85,86] Therapy adherence [19,87] Online support group [86] Sleep apnea management [88]	

^a^AF: atrial fibrillation.

**Table 2:** The number of studies considering user experiences in screening, detection, and management of AF^a^ across the phases of complex interventions [34].

	Identification, n	Development, n	Feasibility, n	Evaluation, n	Implementation, n
Screening	2	1	7	1	3
Detection	5	1	23	0	0
Management	4	3	21	0	0

^a^AF: atrial fibrillation.

### User Experience and Behavioral Approaches in Phases of Designing Complex Interventions

The various methods used by the selected papers for each phase of the UK MRC framework for the design of interventions [34] are shown in Table 3 and described below.

**Table 3:** The methods used to understand user experiences and behavioral approaches in the different phases of developing and evaluating complex interventions.

Phase	Methods
Identification	Survey [72,73,79,87,89,90] Interviews [65,75,86,87,91]
Development	Discussions [52,61,87] Focus groups [61,81]
Feasibility	Survey [17-19,38,39,49,54-57,59-64,66,67,74,76,77,80,82,84,85,87,88,92-96] Adherence [14,18,19,38,39,52,55,56,61,64,66,68,69,74,81,84] Interview [13,18,19,50,52,54,58,62,67,68,71,74] Observation [39,52,58,67]
Evaluation	Interviews [52,53] Observation [52]
Implementation	Interviews [51,70] Adherence [51,53,70]

### Identification Phase

Studies elicited in the review aimed to understand the attitudes of stakeholders toward mHealth interventions for AF, including the perceived advantages and disadvantages, and case scenarios using surveys and interviews [73,75]. While most studies considered micro level behavior, Waring et al [86] explored the interests of older patients with AF in meso level online community support groups. Most of the surveys and questionnaires were not validated, custom-made, and not designed together with users and stakeholders. Although Ding et al [91] and Nuvvula et al [89] reported that they prepared their survey iteratively by consulting a review panel or “experts,” no further elaboration was provided. Reading et al [65] based their interview questions on the Unified Theory of Acceptance and Use of Technology model, where engaged and unengaged users were identified based on device use data. Patients showed an active interest in all interventions barring online support groups, which were more likely to be used by those who were already using web-based tools [86]. Participants with diagnosed AF were more likely to share their data with providers compared to those with undiagnosed AF [89]. Cher et al [75] reported discord between the interests of physicians and patients: physicians wanted a tool that could support their workflow while patients valued tracking and logging their health data. Boriani et al [72] reported that 70% of physician participants in their study were not willing to initiate mass screening using wearables in their current state, and in over half of the cases, the use of wearables was the patients’ personal decision. In Ding et al [91], over half of the respondents did not use wearables due to a lack of clinical and other guidelines on the same. In Manninger et al [73], most physicians believed that the costs of wearables should be shared between patients and insurers. Electrophysiologists were more likely to recommend wearable devices compared to general physicians [72,91]. Physicians preferred handheld, single-lead ECG devices over PPG and other devices [72,73]. On the whole, there can be varying and conflicting preferences among users and stakeholders, which need to be considered in identifying suitable mHealth technology in the design of interventions.

### Development Phase

Four studies considered a user-centered approach to developing interventions. Dickson et al [61] and Toscos et al [81] proposed focus groups to ascertain user needs and gaps. In Dickson et al [61], selected patients and caregivers along with developers were also invited to a hackathon to improve the usability and interactivity of the smartwatch intervention for detecting AF. Orchard et al [52] developed their initial screening program theory based on previous discussions with doctors, nurses, and researchers. Peleg et al [87] iteratively designed the graphical user interface of their mobile apps by consulting academic and industrial experts and group members, although they did not elaborate on their methods. They used the transtheoretical model to stratify users based on their existing behavior into precontemplation, preparation, and action stages based on their readiness to change with respect to therapy compliance. Patients in the precontemplation stage received encouraging messages once they started using the behavior change app, acknowledging their willingness to get started with therapy. In Toscos et al [81], patients felt that they received inadequate information and support from doctors and health care providers. As a result, Toscos et al [81] developed an app that provided up-to-date information in simple language to patients by both electronic and print means. In Orchard et al [52], health care workers were provided with integrated electronic tools that can automatically identify eligible patients and financial incentives along with the ECG devices to overcome time constraints and the lack of reimbursement policies.

### Feasibility Phase

The majority of the studies (43/55, 78%) considered user experiences and behavior of the interventions in the feasibility stage as a part of the progression criteria through questionnaires, interviews, observation, and adherence to interventions. Questionnaires and surveys typically considered ease of use, attractiveness, novelty, and concerns [17,56,85]. Among questionnaires that assessed user satisfaction and the usability of interventions, some studies have used standardized scales, such as the System Usability Scale [61,62] and the Mobile Application Rating Scale [92], while others have used custom ones with similar Likert scale questions [64]. Adherence to an app or a device was measured as the number of days it was used or the number of signals that were transmitted. Nguyen et al [49] used repeated surveys to track the usability of their intervention across a period. Only one study [93] assessed the impact of an intervention on users’ activities of daily living such as moving, sleeping, eating, and using the restroom.

However, some studies asked leading questions in their surveys. For example, in Mcmanus et al [59], some of the survey questions included “The app gives me reassurance” and “This app will improve my general well-being.” Qualitative interviews and observation studies were necessary to obtain an in-depth understanding of user perspectives and experiences that surveys and log data with predetermined outcome measures could not do. In the study by Ding et al [62] that assessed the use of a smartwatch for AF monitoring after stroke, patients expressed the need for both continuous monitoring and in the form of a passive system that did not require extensive engagement. In Peleg et al [87], which involved an app to increase patient compliance, interviews confirmed the consistency between participants’ and authors’ perceptions of “patient engagement.” Macniven et al [54] and Ding et al [91] stated that they co-designed interviews and outcome measures together with stakeholders, although they did not provide a detailed description of the same. In Gawalko et al [57] and Nathania et al [18], reminders and positive feedback on the use of heart monitoring devices were used to increase the usability of these interventions. Nathania et al [18] based their surveys and interviews on the Technology Acceptance Model and examined the perceived usefulness, perceived ease of use, and behavioral intent with regard to the self-management app. Participants found the ability of the app to improve AF knowledge, management, and clinical consultations to be useful and felt that having it on their personal devices would improve the ease of use. Most participants were willing to continue using it, although actual use data showed skewed interest for different features, that is, preference for AF-related content and decreased interest for motivational messages and reminders over time. Wong et al [67] used observation studies, where the time taken for physicians to place the electrodes of a handheld ECG device and obtain the signal was considered as a study outcome. Only Desteghe et al [17] considered outcomes at the meso community level in their study on grandparent-grandchildren relationships. A gamification strategy was used to develop a medication reminder mobile app, where the grandparents, who were the patients, and their grandchildren were given rewards on the completion of certain tasks. The grandparents had to take their medicine while the children chose to perform a health-promoting activity such as eating fruits. The rewards were fun activities that both could perform together such as going on a trip.

### Evaluation Phase

There was only one study that focused on this stage of mobile AF health interventions. The paper by Orchard et al [52] on screening used the realist evaluation framework that considers the context (C), mechanism (M), and outcome (O) of interventions. Outcomes are effects resulting from the conditions created by mechanisms operating in a context. Contexts included the general practice regulations; mechanisms that lead to success were practice wide engagement and user acceptance; and outcomes related to motivations, behavior change, and barriers to screening. Along with semistructured interviews, they observed health care workers during their practice and noted the different barriers to screening, attitudes to screening, and motivation to screen in rural and metropolitan general practice community settings. Efficient leadership, regular updates, and defined protocols were found to be crucial for the success of screening interventions.

### Implementation Phase

Studies proposed semistructured interviews and adherence to intervention implementation in understanding the acceptability, barriers, and facilitators of interventions in different community contexts [51,70]. In Lowres et al [70], which examined the uptake of a public AF screening program in pharmacies, increasing the customer’s confidence regarding the ability of the pharmacist ability to use the device was crucial for their participation. The unavailability of Wi-Fi networks and problems with mobile phone reception also challenged customer recruitment. A combination of advertising using flyers and posters and directly approaching customers was necessary to maximize recruitment. Furthermore, pharmacists reported conflicts between their regular work, that is, dispensing drugs and screening especially during busier times. Combining AF screening with other screening services, such as blood pressure and sugar monitoring, resulted in more time-efficient workflows.

## Discussion

### Principal Findings

While at the outset, it was hoped to ascertain quantitative evidence for the effectiveness of different types of mHealth interventions and their impact on patient’s lives, experiences, and patient outcomes; instead, the information presented in the papers reviewed lent itself qualitatively to assess the need and effectiveness of the methods used to elicit user experiences and device usability. The different methods used to study user experiences and behavior with AF mHealth interventions included interviews, surveys, questionnaires, device data, focus groups, hackathons, and observation. Some were custom-made while others were prevalidated, such as the System Usability Scale and Mobile Application Rating Scale, or based on preexisting models, such as the Technology Acceptance Model. In the identification phase, studies showed that there can be considerable differences between the needs of patients and health care workers. In development, user perspectives guided the development of the device and apps, and in providing technical and nontechnical support to ensure the intervention’s success. Although several papers considered usability as an outcome in feasibility studies, the interviews, surveys, and other instruments were not designed together with direct users and other stakeholders. Studies using interviews provided a richer understanding of user experiences and perspectives. The evaluation and implementation phase studies entailed unique real-world challenges associated with user participation, acceptance, and workflows that could not be captured by studies in the previous phases.

There was only a single study that specifically mentioned the use of behavioral change approaches in the design of interventions [87]. While the rest of the papers entailed approaches that could be categorized and mapped to different levels of sociotechnical health care systems, the studies did not regard the approaches as behavior-driven or behavioral change approaches in their study objectives. Most of the studies focused on user behavior at the micro level, with none reporting behavior change at the macro level. Furthermore, the long-term applicability of the used methods is unclear. Feedback-based [18,57,87] and goal-based approaches [17,87] have been found to be applicable in the long term in other contexts, although 3 of the selected studies did not entail a variation over time across the respective study durations of 28 weeks [57], 6 weeks [18], and 12 weeks [17]. These approaches require a temporal variation to ensure their long-term applicability, as across time and changing contexts, the same kind of feedback may fail to elicit new behavior, and previous goals may no longer seem beneficial or achievable [43]. In this regard, Peleg et al [87] moved patients between the stages of the transtheoretical model of health behavior change based on their engagement with the app and completion of tasks, even though the authors did not specify the exact duration of their preliminary assessment study.

This systematic review linked studies to different phases of the MRC framework for the design of complex interventions, which was missing in reviews to date. It elucidated the various methods used to understand and influence user experiences and their strengths and drawbacks. It highlighted the lack of emphasis on behavior change and behavior-driven methods, as well as their limited long-term applicability. The results showed that, in most of the studies and especially in the feasibility phase (n=43), the measures for assessing user experiences were based more on authors’ own perceptions of an intervention’s success and usability and less on users’ journeys, goals, and lived experience in health care systems. Going forward, studies aimed at understanding collective behavioral trends at the societal or macro level, which have been unexplored by studies to date, would need to account for political and socioeconomic health system factors such as public approval and government policies [47].

### Limitations

This review had some limitations. Only studies in English were considered. The statistical validity of the study outcomes and the existence of biases were not analyzed due to the lack of studies. In addition, some studies were incomplete and only their methodology could be assessed.

### Conclusions

This systematic review proposed the novelties of mapping, first, studies of different phases of the MRC framework for the design of complex interventions, and second, behavioral approaches to different levels of health care systems. All in all, most studies to date have not attempted to obtain a deeper understanding of user experiences and related behavior. The challenges associated with introducing mHealth interventions in sociotechnical health care systems, which impact the uptake and usability of mHealth interventions [36,97] and impede the health care system’s ability to help users achieve their goals in life [29,30], have not been adequately addressed. The results of this review emphasized the need for a change in the direction of AF mHealth research to focus more on user experiences and behavior. Such a change would be possible through co-designing through life with patient and public involvement and using systems approaches that consider people with complex health needs, for example in the context of multiple long-term conditions. Our analysis of the different works to date with respect to user experiences and behavior serves as a starting point for future research using mHealth interventions to improve the lives and journeys of patients with AF and their wider network of formal and informal carers.

## Appendix

Multimedia Appendix 1 PRISMA (Preferred Reporting Items for Systematic Reviews and Meta-Analyses) checklist.

## Abbreviations

AF: atrial fibrillation
ECG: electrocardiography
ISO: International Organization for Standardization
mHealth: mobile health
MRC: Medical Research Council
PPG: photoplethysmography
PRISMA: Preferred Reporting Items for Systematic Reviews and Meta-Analyses
